# Editorial: Vascular dysfunction beyond pathological pregnancies. An international effort addressed to fill the gaps in Latin America, Volume II

**DOI:** 10.3389/fphys.2022.989407

**Published:** 2022-08-31

**Authors:** Carlos Escudero, Fernanda Regina Giachini, Reggie García-Robles, Carlos Galaviz-Hernandez, Alicia E. Damiano

**Affiliations:** ^1^ Vascular Physiology Laboratory, Department of Basic Sciences, Faculty of Basic Sciences, University of Bío-Bío, Chillán, Chile; ^2^ Group of Research and Innovation in Vascular Health (GRIVAS Health), Chillán, Chile; ^3^ RIVATREM - Red Iberoamericana de Alteraciones Vasculares en Transtornos del Embarazo, Chillan, Chile; ^4^ Institute of Biological Sciences and Health, Federal University of Mato Grosso, Barra do Garças, Brazil; ^5^ Departamento de Ciencias Fisiológicas, Facultad de Medicina, Pontificia Universidad Javeriana, Botogá, Colombia; ^6^ Academia De Genómica, Instituto Politécnico Nacional-CIIDIR Durango, Durango, Mexico; ^7^ Laboratorio de Biología de la Reproducción, Instituto de Fisiología y Biofísica Bernardo Houssay (IFIBIO)- CONICET- Facultad de Medicina, Universidad de Buenos Aires, Buenos Aires, Argentina; ^8^ Cátedra de Biología Celular y Molecular, Departamento de Ciencias Biológicas, Facultad de Farmacia y Bioquímica, Universidad de Buenos Aires, Buenos Aires, Argentina

**Keywords:** placenta, pregnancy complications, reproductive health, vascular endothelium, editorial

Recent evidence suggests that vascular changes associated with pregnancy complications, such as preeclampsia; gestational diabetes; growth restriction; autoimmune diseases; among others, affect the function of the maternal and offspring vascular systems after delivery and may be extended until adult life (Giachini et al., 2017; Dayan et al., 2018; Honigberg et al., 2019). Furthermore, since the vascular system contributes to systemic homeostasis, defective development or function of blood vessels predisposes both mother and infant to future risk for chronic disease (Davis et al., 2012; Phipps et al., 2019).

In Latin American countries, the rate of morbi-mortality due to pregnancy complications and cardiovascular diseases has a higher incidence than in high-income countries (HIC) (Lopez-Jaramillo, 2009; Cuevas et al., 2014). But, the biomedical investigation into the cardiovascular consequences of pregnancy coming from Latin American countries still falls short of what would be expected considering the magnitude of those diseases. Although there are obvious deficiencies in terms of economies and scientific infrastructure between HIC and Latin American countries, strength in terms of scientific productivity in this field could be underestimated. Which is associated with language limitations and publication in journals not indexed in major citation databases resulting in low-impact publications (Van Noorden, 2014). Unfortunately, an investigation performed in LIC or MIC receives minor citation even if it is published in high-impact journals (Meneghini et al., 2008). As a result, we could speculate that potentially unique features of vascular disease associated with pregnancy complications can be unnoted in the global scientific community.

Trying to overcome this potential bias in the literature, in collaboration with the Red Iberoamericana de alteraciones Vasculares Asociadas a TRastornos del EMbarazo (RIVA-TREM), we generate the first volume of our Research Topic in Frontiers in Physiology. We published 13 articles, with the valuable contribution of 60 authors. Since then, our scientific production has been viewed more than 60K times**,** and an increasing number of downloads from researchers worldwide. Then, we decided to continue encouraging Latin American Researchers in vascular biology to continue contributing to a better understanding of vascular dysfunction associated with pregnancy diseases and show the gaps in the literature, to overcome this hidden effect of our scientific production. Therefore, it is a pleasure to present volume II, with more authors (n = 121) and manuscripts (n = 19) than volume I. In the following sections, we highlight the relevance of those publications (Blanco et al., 2022).

One important event that massively affected the Latin American countries and the entire world was the pandemic of COVID-19. In this issue, Ayala-Ramirez et al. (10.3389/fphys.2022.785274) carry out a broad and concise review of the evidence about the compromise in pregnancy due to SARS-CoV-2 infection and COVID-19. In addition, they analyze alterations in cardiovascular adaptations and changes in the renin-angiotensin-aldosterone system during pregnancy. Authors emphasize information on pregnant women the information on pregnant women in Latin America and the need to generate more excellent knowledge about the impact of the pandemic on pregnancy in these countries.

The concern about the impact of non-transmissible maternal conditions on pregnancy and offspring, such as obesity and diabetes mellitus, has also been carefully considered by the contributors’ authors. In this regard, gestational diabetes mellitus is a complex multifactorial disorder with a strong genetic predisposition. Higa et al. (10.3389/fphys.2021.760251) reviewed the effect of gestational diabetes mellitus as a trigger of fetal programming associated with a high risk of metabolic and cardiovascular diseases. Since the high diabetes prevalence before and during pregnancy and the increasing prevalence of obesity in pregnant women, the programming effect of those conditions extends the awareness for the next generations. This timely review provides information on studies of both human and experimental models addressing putative mechanisms involved in the fetal programming of the heart damage associated with maternal diabetes. In addition, Ortega-Contreras et al. (10.3389/fphys.2022.769924) describe different single nucleotide variations on genes related to different pathophysiological mechanisms associated with gestational diabetes mellitus. This analysis includes genes associated with alterations in transcription factors, hormones, membrane proteins, enzymes, growth factors, and others that may disturb insulin production and excretion or impair/modulate endothelial function.

Genetic factors and other parameters, such as hormones, are involved in maternal diabetes and placental dysfunction mechanisms. Louis et al. (10.3389/fphys.2021.76592) present original data on how the melatonin hormone prevents oxidative stress in maternal blood and placenta of women with diabetes. The authors found that melatonin levels were higher in maternal blood but reduced in the placental villus of women with type 2 diabetes. These findings were associated with a high rate of placental superoxide release, as well as in blood mononuclear cells of diabetic women, which was significantly reduced by melatonin. In addition, melatonin reduces placental and peripheral mononuclear cells’ apoptosis. In addition, in the field of melatonin, Valenzuela-Melgarejo et al. (10.3389/fphys.2021.767684) evaluated the evidence for the potential beneficial effects of this molecule during hypertension. These authors describe that melatonin supplementation reduces blood pressure levels, prevents oxidative stress, improves antioxidant systems, and reduces Soluble Fms-Like Tyrosine Kinase 1 (sFlt-1) levels. Hence, melatonin can prevent endothelial damage in the placenta and may restore umbilical and uterine blood flow affected in pathological pregnancies, including preeclampsia or gestational diabetes.

Araujo-Silva et al. (10.3389/fphys.2021.701767) showed that maternal diabetes and obesity modulate immunological and metabolic changes associated with an increased risk of growth and development abnormalities in offspring in a murine model. These results are of great interest due to the possibility of being translated to human pregnancy.

Another essential contributor to maternal morbidity within the Latin American population is preeclampsia. The pathophysiology of preeclampsia and its complications are still elusive. In this field, Ayala-Ramirez et al. (10.3389/fphys. 2021.708824) reported an increase in pro-apoptotic molecules, such as Fas ligand (FasL) and TNF-Related Apoptosis-Inducing Ligand (TRAIL). These augmentations were observed in extracellular vesicles derived from cultures of placental explants from pregnancy affected by preeclampsia. Also, they showed how these extracellular vesicles had a high capacity to induce apoptosis in an *in vitro* model. This study explored how placental-derived extracellular vesicles might participate in the pathophysiology of preeclampsia. The results raise interesting questions about mechanisms involved, like trophoblast, immune and vascular endothelial cells. Whereas, Torres-Vergara et al. (10.3389/fphys.2021.805082) hypothesized that another pro-apoptotic marker, sFlt-1, maybe a protective rather than deleterious on the cerebrovascular bed. They proposed that the elevation of sFlt-1 observed in preeclampsia could have a protective effect on the blood-brain barrier.

Another important aspect is to reveal biomarkers to be used to predict preeclampsia. Corominas et al. (10.3389/fphys.2021.785219) found that serum uric acid levels are increased in all forms of preeclampsia. However, the time of the rise of uric acid levels depended on the severity of the disease. In addition, their results revealed that uric acid levels do not increase in normotensive gestations with intrauterine growth restriction (IUGR), allowing a differential diagnosis between IUGR with and without preeclampsia. They found that uric acid levels less than 1.5 are a helpful parameter with a substantial exclusion value and high sensitivity for women who are not expected to develop preeclampsia. Implementing this low-cost test as a routine medical practice for all pregnant women would improve the use of resources in low-income countries. A more sophisticated analysis describes that microRNAs also have potential use as a biomarker for preeclampsia. Luizon et al. (10.3389/fphys.2021.678184) identified as upregulated the miR-204-5p in second-trimester plasma samples of women who eventually developed preeclampsia. Also, they showed through bioinformatics tools how miR-204-5p regulates the expression of genes potentially linked to preeclampsia. Therefore, they propose this microRNA as a biomarker of preeclampsia.

Several contributions are included in this issue regarding the pathophysiological mechanisms by which preeclampsia elicits trophoblastic cell and placental dysfunction. Medina et al. (10.3389/fphys.2021.774095) showed that aquaporin (AQP)-9 can function as a lactate transporter and have a role in energy metabolism or as a reactive oxygen species (ROS) scavenger. Authors propose that the lack of functionality of AQP9 may impair the placenta’s lactic acid utilization, promoting ROS accumulation and adversely affecting the survival of the trophoblast cells. It also confirmed the presence of two mitochondrial subpopulations, which exhibit different morphologic and metabolic states, and revealed that AQP9 localized in the heavy/large mitochondria of the villous trophoblast cells.

Hence, other AQPs might be enrolled in a range of trophoblastic/placental activities during pregnancy. Dos Passos Junior et al. (10.3389/fphys.2021.696495) investigated the role of AQP-3 in trophoblastic cell migration. They show that tumor necrosis factor α (TNF-α) negatively modulates AQP-3 in placental explant and trophoblastic cells, reducing cell migration. Yet, they used an experimental model of spontaneously hypertensive rats (SHR) to show that placental levels of TNF-α were higher in the hypertensive group, whereas AQP-3 expression was reduced.

In addition, Troiano et al. (10.3389/fphys.2021.760237) used SHR rats to investigate the role of caveolae/caveolin-1 (Cav-1) on endothelial nitric oxide synthase (eNOS) activation during pregnancy. They found that in normotensive and hypertensive pregnancy, eNOS activation augment due reduced Cav-1/eNOS interaction favoring nitric oxide production in the aorta and mesenteric resistance arteries.

Mendes Silva et al. (10.3389/fphys.2021.766382) showed original contribution in a scientific area not yet analyzed in detail in Latin American countries, such as the infection of Group B *Streptococcus* (GBS) during pregnancy and its negative consequences on the function of trophoblast and endothelial cells. They use an *in vitro* approach in which chorionic villi explant, HTR-8/SVneo trophoblastic cell, and Ea. hy926 endothelial cell line were exposed to non-infected GBS. Also, they treated those cells with pentacyclic triterpene uvaol (urs-12-ene-3,28-diol), a component of the oliva oil with anti-inflammatory effects. Their findings demonstrated that GBS causes placental inflammation and oxidative stress, reducing trophoblast invasion of endothelial cells and increasing CXCL-8 and IL-6. These key factors participate in vascular dysregulation observed in several diseases. Since uvaol treatment prevented most of the GBS-provoked changes, they suggest that this molecule may be prophylactic for the potentially harmful effects of GBS infection for both the mother and the fetus.

Transmissible infections impose a maternal stress environment during pregnancy. Maternal stress refers to a set of chronic or acute injuries experienced by the mother during the pregnancy. One recognized model of maternal stress is the early alcohol consumption during pregnancy, a condition related to the development of fetal alcohol syndrome spectrum. The mechanism can be directly associated with fetal damage or indirectly through placental dysfunction. Gualdoni et al. (10.3389/fphys.2021.815760) provide bibliographic evidence of the harm imposed on placental structure and function secondary to maternal alcohol consumption. The authors propose a mechanism by which alcohol can disrupt the vascular development of mouse placenta by impairing vascular endothelial growth factor (VEGF) and its downstream effectors such as eNOS and metalloproteinases.

Costa et al. (10.3389/fphys.2022.787617) bring to light that the immune system might be bridging maternal stress and offspring vascular dysfunction. They acknowledge a disrupted modulation of the immune system by the maternal hypothalamic-pituitary-adrenal axis rise in response to maternal stress. In response to maternal stress, monocytes, natural killer cells, T cells, B cells, and antigen-presenting cells can be affected, contributing to the immune response-induced vascular dysfunction. This is characterized by an enhanced renin-aldosterone-angiotensin system, reactive oxygen species (ROS) accumulation, and toll-like receptor activation.

Maternal immune system dysfunction is also related to antiphospholipid syndrome (APS), an autoimmune disorder that is characterized by pregnancy morbidity or thrombosis and persistent antiphospholipid antibodies (aPL). These antibodies can bind to the endothelium and induce endothelial activation, which is evidenced by the expression of adhesion molecules and the production of ROS and subsequent endothelial dysfunction marked by decreased synthesis and release of nitric oxide. Velasquez et al. (10.3389/fphys.2021.764702) revealed differences in endothelial activation and dysfunction among the different groups of patients with APS, which should be considered in future studies to evaluate new therapies, especially in refractory cases. On the other hand, Rodriguez et al. (10.3389/fphys.2021.706743) studied the impact of aPL from different patient populations on endothelial cell mitochondrial function, activation of the mTOR and autophagy pathways, and cellular growth. They found that aPL induces cellular stress evidenced by mitochondrial hyperpolarization and increased activation of the mTOR and autophagic pathways, which may play a role in the pathogenesis of obstetric APS.

Finally, Accialini et al. (10.3389/fphys.2021.667367) demonstrated that anandamide, a member of the endocannabinoid system, exerts a differential effect on prostaglandins concentration and NO bioactivity. This regulatory system is differentially involved in the onset of labor depending on the type of delivery, vaginal or cesarean section.

Therefore, we encourage the reader to analyze in detail the evidence in those manuscripts, and we invite you to discuss further, criticize, replicate results and learn from Latin American researchers. This invitation would help spread scientific research from this part of the world and overcome scientific bias in the international literature with extensive evidence from HIC.
